# Drug-Eluting Biopsy Needle as a Novel Strategy for Antimicrobial Prophylaxis in Transrectal Prostate Biopsy

**DOI:** 10.1177/1533034617722080

**Published:** 2017-08-02

**Authors:** Marcin Sieczkowski, Artur Gibas, Andrzej Wasik, Agata Kot-Wasik, Lidia Piechowicz, Jacek Namieśnik, Marcin Matuszewski

**Affiliations:** 1 Department of Urology, Medical University of Gdańsk, Poland; 2 Department of Analytical Chemistry, Gdańsk University of Technology, Gdańsk, Poland; 3 Department of Microbiology, Medical University of Gdańsk, Gdańsk, Poland

**Keywords:** prostate biopsy, prostate cancer, biopsy needle, antibiotic prophylaxis, *Escherichia coli*

## Abstract

**Objectives:**

To preclinically evaluate drug-eluting biopsy needles (patent pending WO2016118026) as a new potential way of antimicrobial prophylaxis for transrectal prostate biopsy.

**Methods:**

Twenty steel biopsy needles have been coated with polyvinyl alcohol, ciprofloxacin, and amikacin. Modified biopsy needles have been randomly divided into 3 groups (1:2:1 ratio). Needles from group I were immersed for 30 minutes in dedicated test tubes containing saline. Needles from group II were immersed (one by one) for 5 seconds in a set of 12 test tubes containing saline. Then, each solution was analyzed using high-performance liquid chromatography. The results were compared with the susceptibility break points for *Escherichia coli*. Group III was incubated with *E coli* strains on Mueller-Hinton plate and then the bacterial inhibition zones surrounding needles were measured.

**Results:**

The average concentration of antibiotics eluted from needles (group I) was 361.98 ± 15.36 µg/mL for amikacin and 63.87 ± 5.95 µg/mL for ciprofloxacin. The chromatographic analysis revealed the gradual release of both antibiotics from needles (group II). The concentration of amikacin released from needles exceeded the break-point value from first to ninth immersion. Ciprofloxacin concentration was higher than break-point value in all immersions. The average bacterial inhibition zone minor axis was 42 ± 5.7 mm (group III).

**Conclusions:**

The use of drug-eluting biopsy needle could be a new potential way of antimicrobial prophylaxis for transrectal prostate biopsy. This study confirmed its biological activity as well as the gradual release of antibiotics from its surface. Confirmation of its preventive role, in terms of infectious complications after transrectal prostate biopsy, has to be evaluated in a clinical trial.

## Introduction

Transrectal ultrasound-guided prostate biopsy (TRUS-Bx) is a standard method for histological diagnosis of prostate cancer and one of the most commonly practiced urological procedures in the world.^1^ It is estimated that there are more than 800 000 TRUS-Bx performed every year in the United States alone.^2,3^ Due to transrectal approach and multiple sampling, TRUS-Bx is associated with up to 7% risk of infectious complications such as urinary tract infections, prostatitis, epididymitis, or even severe sepsis and septic shock.^4^ Therefore, there is an essential need for periprocedural antimicrobial prophylaxis which is indicated in all patients undergoing TRUS-Bx, with the best evidence among the urological procedures.^5–7^

European Association of Urology and American Urology Association guidelines on TRUS-Bx antimicrobial prophylaxis stated that oral fluoroquinolones are the first-line prophylactic agents.^1,8^ However, in the past few years, an increased resistance of rectal flora to fluoroquinolones associated with a rise in severe infectious complications has been reported.^3,9^ The main pathogen responsible for this phenomenon is fluoroquinolone-resistant *Escherichia coli*, which causes most postprocedural sepsis episodes.^10^ Rapidly growing literature on this issue showed a large percentage (>20%) of those strains present in rectal flora of patients undergoing TRUS-Bx.^2,11^ This means that significant proportion of patients do not receive effective antimicrobial prophylaxis prior to prostate biopsy.

The existing methods to reduce the rate of prostate biopsy–related infections include transperineal prostate biopsy; different regimens of oral, intramuscular, and intravenous antimicrobial prophylaxis; or targeted antimicrobial prophylaxis which requires rectal swab sampling before TRUS-Bx.^11,12^ All of these methods have some disadvantages that result in their limited use.^11,12^ The transperineal prostate biopsy is a more complicated and painful procedure and requires a general anesthesia. Until now, none of various empiric antibacterial prophylaxis regimens that have been proposed did not become the standard over fluoroquinolones and the choice of proper one remains debatable. Even targeted antimicrobial prophylaxis, which is a promising method, still needs more research concerning its efficiency.^11^

The invention presented below represents a novel approach to the problem of TRUS-Bx-related infectious complications. It consists in creating a polymer-coated biopsy needle that releases the drugs directly to the prostate during the procedure. This solution may allow the coadministration of various antibiotics, thereby broaden their spectrum of activity and potentially reduce the number of infectious complications.

The aim of this study is to preclinically evaluate drug-eluting biopsy needle (DEBNs; patent pending WO2016118026) as a new potential way of antimicrobial prophylaxis for TRUS-Bx.

## Methods

Twenty standard steel biopsy needles (FastCutP MGP 1620, AISI 304, 16 G × 200 mm; MDL, Delebio, Italy) have been used in the following experiments. Each needle consisted of cannula, stylet, and biopsy gun holder, which was coated according to the procedure described below.

### Biopsy Needle Coating

The 5% aqueous poly(vinyl alcohol) (PVA, Mowiol 18-88; Sigma-Aldrich, St Louis, Missouri) solution containing active ingredients, amikacin (Polpharma, Starogard Gdański, Poland) and ciprofloxacin (Gedeon Richter, Grodzisk Mazowiecki, Poland), was prepared for coating the biopsy needle. The surface of each biopsy needle cannula was cleaned with fine sandpaper (no. 4000) and then subjected to electrochemical etching (connected as the anode [−]) for 15 seconds in an aqueous solution of hydrochloric acid (1:1, vol/vol). After thorough rinsing with distilled water and shaking off excess water, the distal part of each cannula (5 cm long) was immersed in the PVA solution and dried in a stream of warm air. The coating and drying process was repeated once more and the needle was covered with cap and stored in the fridge before further application.

After the coating process, 20 DEBNs have been randomly separated into 3 groups in a 1:2:1 ratio. The first (I, 5 needles) and second groups (II, 10 needles) were analyzed by high-performance liquid chromatography (HPLC) technique in “coating method repeatability test” (I) and in “injection simulation test” (II), respectively. The third group (III, 5 needles) was dedicated for bacterial growth inhibition test.

### High-Performance Liquid Chromatography Technique

Separation of amikacin and ciprofloxacin was performed using Titan UHPLC, 1.9 μm, C18, 100A (100 mm × 2.1 mm) column working at 40°C. Mobile phase was consisted of acetonitrile and water, both containing 1% vol/vol of formic acid. Flow rate of 0.3 mL/min was used, resulting in a short analysis time (2.5 and 4.3 minutes for amikacin and ciprofloxacin, respectively).

An Agilent 1200 Series Rapid Resolution LC system (Santa Clara, California) consisting of an online degasser, a binary pump, a high-performance SL autosampler, a thermostated column compartment, and a photodiode array diode-array detector (DAD) detector has been used for analytical performance. The system was coupled with a Q-Trap 4000 triple quadrupole mass spectrometer from Applied Biosystems (Foster City, California). All data were collected and processed using Analyst 1.5.2 Software.

Q-Trap 4000 triple quadrupole mass spectrometer (AB SCIEX, Framingham, MA) with electrospray ionization source working in positive ion mode was applied for quantitative analyses. Optimization of tandem mass spectrometry (MS/MS) conditions (MRM [multiple reaction monitoring] mode) was done using solution containing 1 μg/mL of antibiotics.

### Coating Method Repeatability Test

The experiment has been performed to assess the repeatability of coating process in terms of the amount of antibiotics eluted from DEBNs. Five DEBNs (group I) were immersed for 30 minutes in dedicated tubes (1 needle–1 tube) containing 6 mL of NaCl solutions (0.9% m/m). The experiment was performed at 37°C in a water bath. Afterward, in case of amikacin analysis, 10 µL of each solution was diluted to 1 mL with deionized water. Further, these solutions were analyzed using HPLC-MS/MS as described above. In case of ciprofloxacin analysis, 40 µL of each solution was diluted to 1 mL with deionized water and analyzed with the same method.

### Injection Simulation Test

The second test was called injection simulation. This experiment has been done to mimic multiple injections during TRUS-Bx. Ten sets of 12 tubes containing 6 mL of NaCl solution (0.9% m/m) were prepared and immersed in a water bath thermostated at 37°C. Each DEBN from the second group was immersed for 5 seconds in tube #1 from a dedicated set. Next, the same DEBN was immersed for 5 seconds in tube #2. This procedure was repeated for the remaining tubes (#3 to #12). Finally, 1 mL of the solutions, from all tubes and sets, were taken for HPLC-MS/MS analysis to determine the concentrations of amikacin and ciprofloxacin, according to the method described above. The obtained results were compared with the MIC susceptibility break points for *E coli* (S) established by the European Committee on Antibiotic Susceptibility Testing (EUCAST).^13^ For analyzed antibiotics, these values are: 8 mg/mL for amikacin and 0.5 mg/mL for ciprofloxacin.

### Bacterial Growth Inhibition Test

The third experiment was designed and conducted using the diffusion method in accordance with the EUCAST criteria.^14^ Mueller-Hinton agar (Becton Dickinson, Warsaw, Poland) plate, 4 mm in depth, was used in antimicrobial susceptibility testing.

*E coli* (ATCC 25922) strain was grown in blood agar base for 18 hours at 37°C, then suspended in saline to reach 0.5 on the McFarland scale (1-2 × 1.5 × 10^8^ CFU [colony-forming units]/mL). Sterile swab was dipped into the bacterial suspension and inoculated onto agar plate. Then, each DEBN (from group III) was placed on this surface and incubated for 18 hours at 35°C. The results were evaluated visually by observing the appearance or absence of zones of bacterial inhibition surrounding DEBN. The minor axis of bacterial growth inhibition zones was measured.

## Results

**Figure 1.**
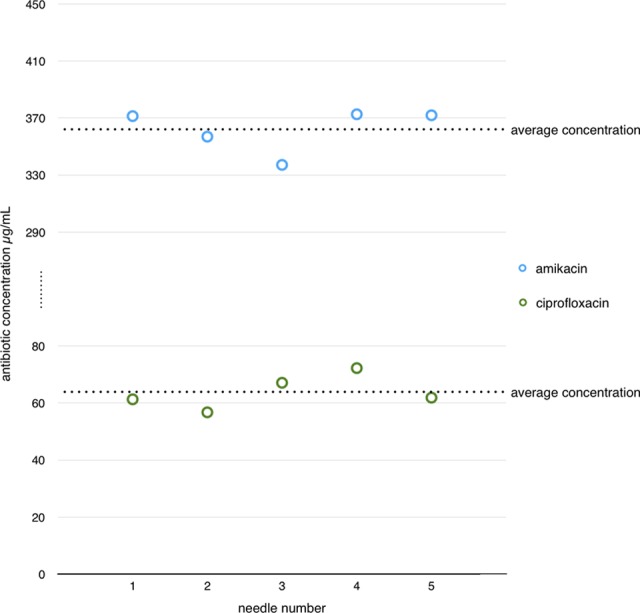
Coating method repeatability test (group I).

The average concentration of antibiotics eluted from DEBNs (group I) was 362 ± 15.4 µg/mL for amikacin and 63.9 ± 6.0 µg/mL for ciprofloxacin. The repeatability of DEBNs coating process assessed by the coefficient of variation (CV = standard deviation [SD]/mean) was 4.2% for amikacin and 9.3% for ciprofloxacin. The results are shown in Figure 1.

**Figure 2.**
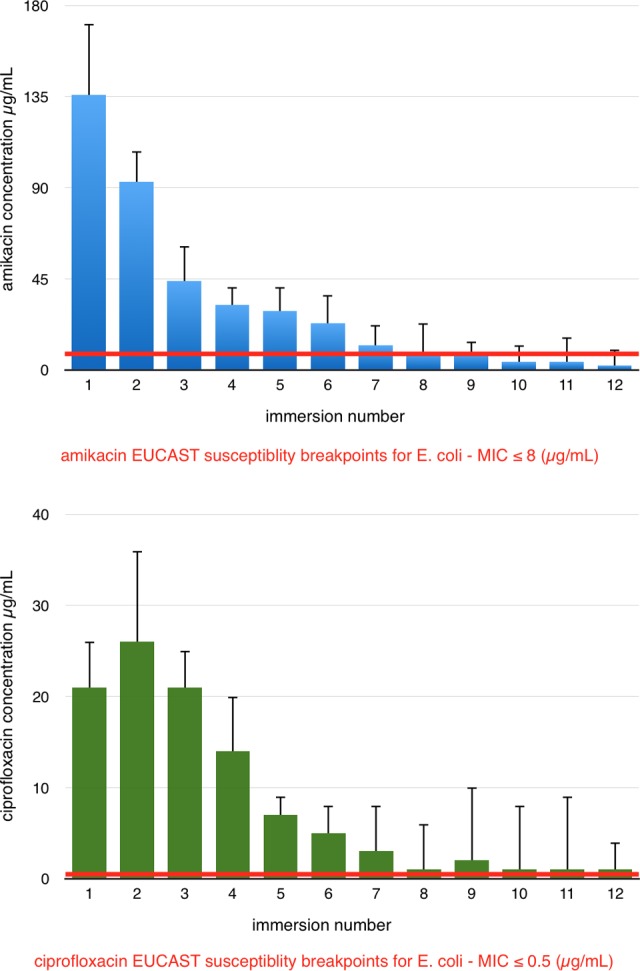
Injection simulation test (group II).

The concentrations of antibiotics released from DEBNs (group II) in subsequent immersions were determined (Figure 2). For immersions 10, 11, and 12, the values of amikacin concentration were between the limit of quantification and the limit of detection.

The HPLC-MS/MS analysis revealed the gradual release of both antibiotics from coating layer in each of the 12 immersions of DEBNs (group II). The dynamics of antibiotic release is illustrated in Figure 2. The concentration of amikacin released from DEBNs exceeded the S value for *E coli* established by EUCAST from first to ninth immersion. Ciprofloxacin concentration was higher than S in all immersions (Figure 2)

**Figure 3.**
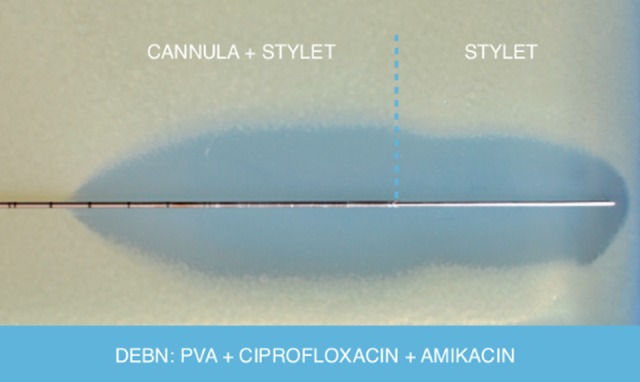
*Escherichia* coli inhibition zone around part of drug-eluting biopsy needle (DEBN) coated with an antimicrobial layer (group III).

Bacterial growth inhibition test showed bacteriostatic activity against *E coli* of all studied DEBNs (group III). In all agar plates, the bacterial inhibition zones appeared as a similar, clear, and oval region around parts of DEBNs coated with an antimicrobial layer (Figure 3). The average bacterial inhibition zone minor axis was 42 ± 5.7 mm (36-49 mm).

## Discussion

The first medical device for controlled release of antibiotics was developed in the 1970s according to Buchholz and Engelbrecht’s idea of releasing antibiotics from the nonbiodegradable polymethylmethacrylate bone cement.^15,16^ Since then many other antibiotic-eluting medical instruments including urinary tract catheters, orthopedic implants, wound dressings, surgical sutures, vascular grafts, or periodontal devices have been successfully introduced.^15^ The main objective of these devices is gradual and slow release of antibiotics in the area of its, usually long-term, application, while DEBN should release drugs directly to the prostate within a short time span of the procedure.

Transrectal intraprostatic drug injections have been studied until now only as a method of treatment for prostatitis and chronic pelvic pain syndrome.^17–20^ Apart from transrectal, also transperineal, transurethral, and even percutaneous suprapubic transvesical route have been used to deliver drugs directly to the prostate gland.^21,22^ The effectiveness of intraprostatic injections of steroids (betamethasone), different types of antibiotics (fluoroquinolones, aminoglycosides), and botulinum neurotoxin type A have been described in the literature.^17–22^ However, due to the lack of randomized control trials, they are not routinely used in this route of administration.^1^

There are no data in the literature on the use of intraprostatic drug injections in antimicrobial prophylaxis prior to TRUS-Bx. In 2013, Issa *et al* published the first focuses on the biopsy needle as a vector of TRUS-Bx-related infections.^23^ Authors described a simple and effective method to reduce the risk of infection after prostate biopsy with formalin disinfection of the biopsy needle after each prostate biopsy core. They found an association between the use of this technique and lower incidence rate of urinary infection and sepsis. However, repeat formalin exposure during prostate biopsy may increase the risk of toxicity and adverse effects.^23,24^

Drug-eluting biopsy needle may allow for the first time to deliver combined antibiotic prophylaxis on surgical instrument during prostate biopsy. This issue could be very important in the era of an alarming trend of an increasing resistance to the standard TRUS-Bx prophylaxis based on fluoroquinolones. The most popular representative of this group is ciprofloxacin, which is still widely used as prophylaxis mainly due to its good prostatic penetration, oral dosage, and safety profile.^25,26^ In 2011, Batura *et al* demonstrated a significant reduction in postbiopsy infectious complications by adding amikacin to ciprofloxacin in TRUS-Bx prophylaxis.^26^ The combination has been proposed based on microbiological analysis that showed high susceptibility of urine and bloodstream isolates to amikacin in patients undergoing TRUS-Bx. However, this combined prophylactic protocol is not commonly used, even though its effectiveness has been confirmed by other authors.^1,27^ One of the main reasons for this is the lack of an oral form of amikacin as well as the need for systemic administration of 2 antibiotics which complicates the prophylaxis and exposes the patient to potential complications.

The broad antibacterial spectrum of combined amikacin and ciprofloxacin prophylaxis as well as attempts of their intraprostatic injections rise to the concept of the DEBN. With this solution, antibiotics released from the surface of the biopsy needle may act in the prostatic tissue contaminated by rectal flora.

Drug-eluting biopsy needles have been coated with PVA. This is a water-soluble synthetic polymer which allows the incorporation and release of ciprofloxacin and amikacin in a relatively short time of TRUS-Bx.

The HPLC analysis revealed that both studied antibiotics were released from DEBNs in physiological saline. Relatively low CV and SD values of antibiotic concentrations released from needles (from group I) may indicate a repeatable methodology of DEBN coating process. For all experiments, only 5-cm-long parts of DEBNs were coated with an antimicrobial layer. This was due to the assumption that the length of a biopsy needle inserted into the prostate tissue usually will not exceed this dimension.

The injection simulation test (group II) showed gradual and stable release of amikacin and ciprofloxacin from DEBNs (Figure 2). However, the dynamics of antibiotic release in saline probably not fully reflects the real situation during TRUS-Bx due to different friction forces. The average concentration rates exceeded the S value for *E coli* established by EUCAST from first to ninth immersion for amikacin and in all immersions for ciprofloxacin (Figure 2). Despite that the average concentration rates of amikacin did not exceed S value in the last 3 immersions, the simultaneous use of amikacin and ciprofloxacin may reduce the number of infectious complications associated with TRUS-Bx. These results provide further support for the high likelihood of therapeutic success of DEBNs.

The above data correlated well with the bacterial growth inhibition test where large zones of *E coli* inhibition surrounding DEBNs (group III) have been observed (Figure 3). Due to the fact that the strain used in experiment (ATCC 25922) is a multidrug-sensitive *E coli* strain (commonly used in quality control study), the inhibitions zones are the result of simultaneous bacteriostatic activity of amikacin and ciprofloxacin. The probable positive effect of such DEBNs for fluoroquinolone-resistant strains was not confirmed. This is a certain limitation of the study. However, it can be expected that all amikacin susceptible strains should also be included in its effect due to the level of amikacin release proved in HPLC analysis.

The expected clinical benefits of DEBN include single-stage drug release during biopsy, combined antibiotic prophylaxis, precise operation in the area of potential infection, smaller doses of administered drugs, and potentially decreased infection rates. This, however, cannot be deduced from the *in vitro* study and needs to be tested in a clinical trial.

The clinical proof of DEBN’s efficacy may be associated also with cost-effectiveness of this solution. Affordable price of coating technology and high costs of management of TRUS-Bx-related infections should be compared with the reduction in infections rates. Adibi *et al* who performed analysis of fluoroquinolones-based versus intensive antibiotic prophylaxis for TRUS-Bx stated that second regimen was substantially more cost-effective even if it is more expensive.^28^

In addition to the use of DEBN as TRUS-Bx antimicrobial prophylaxis, this idea may be used in other applications such as biopsy of other organs or the use of different drugs with, for example, analgesic, antibleeding, or anti-inflammatory activity.

## Conclusion

The HPLC analysis and the bacterial growth inhibition test showed that DEBNs released high concentrations of amikacin and ciprofloxacin and have strong bacteriostatic activity against *E coli*. Adopting this novel strategy for antimicrobial prophylaxis may reduce the rate of infectious complications related to transrectal prostate biopsy. However, the clinical introduction of DEBN requires further studies on stabilization of the active layer and increasing the amount of released drugs during multiple injections. Confirmation of its preventive role, in terms of infectious complications after TRUS-Bx, has to be evaluated in a clinical trial.

## Abbreviations

DEBN: drug-eluting biopsy needle
EUCAST: European Committee on Antibiotic Susceptibility Testing
HPLC: high-performance liquid chromatography
MS/MS: tandem mass spectrometry
PVA: poly(vinyl alcohol)
TRUS-Bx: transrectal ultrasound-guided prostate biopsy
